# Identification of a Novel Antiviral Lectin against SARS-CoV-2 Omicron Variant from Shiitake-Mushroom-Derived Vesicle-like Nanoparticles

**DOI:** 10.3390/v16101546

**Published:** 2024-09-30

**Authors:** Joshua Wiggins, Shazeed-Ul Karim, Baolong Liu, Xingzhi Li, You Zhou, Fengwei Bai, Jiujiu Yu, Shi-Hua Xiang

**Affiliations:** 1 Nebraska Center for Virology, University of Nebraska-Lincoln, Morrison Center 143, 4240 Fair Street, Lincoln, NE 68583, USA; 2 School of Biological Sciences, University of Nebraska-Lincoln, Lincoln, NE 68583, USA; 3 Department of Cell and Molecular Biology, School of Biological, Environmental, and Earth Sciences, University of Southern Mississippi, Hattiesburg, MS 39406, USA; 4 Department of Nutrition and Health Sciences, University of Nebraska-Lincoln, Lincoln, NE 68583, USA; 5 School of Veterinary Medicine and Biomedical Sciences, University of Nebraska-Lincoln, Lincoln, NE 68583, USA; 6 Center for Biotechnology, University of Nebraska-Lincoln, Lincoln, NE 68583, USA

**Keywords:** Shictin, lectin, SARS-CoV-2, shiitake mushroom, extracellular vesicles (EVs), vesicle-like nanoparticles (VLNs), vegetable, exosomes

## Abstract

Lectins are a class of carbohydrate-binding proteins that may have antiviral activity by binding to the glycans on the virion surface to interfere with viral entry. We have identified a novel lectin (named Shictin) from Shiitake mushroom (*Lentinula edodes*)-derived vesicle-like nanoparticles (VLNs, or exosomes) that exhibits strong activity against the SARS-CoV-2 Omicron variant with an IC_50_ value of 87 nM. Shictin contains 298 amino acids and consists of two unique domains (N-terminal and C-terminal domain). The N-terminal domain is the carbohydrate-binding domain (CBD) that is homologous with CBDs of other lectins, suggesting that Shictin inhibits SARS-CoV-2 infection by binding to the glycans on the virion surface to prevent viral entry. This finding demonstrates that exosomes of vegetables are a valuable source for the identification of antiviral lectins. Therefore, it is believed that lectins from vegetable VLNs have potential as antiviral therapeutic agents.

## 1. Introduction

The emergence of a new coronavirus (SARS-CoV-2) in late 2019 caused a global pandemic of severe respiratory infectious disease (COVID-19) and still poses a great threat to public health and the economy [1,2,3]. Although we have vaccines for SARS-CoV-2, developing therapeutics to treat this infectious viral disease is essential. SARS-CoV-2 is an enveloped RNA virus with a positive-sense single-stranded RNA genome of ~30 kb [4]. SARS-CoV-2 entry into cells is mediated by the spike (S) glycoprotein, which is cleaved by Furin protease into S1 and S2 subunits during viral assembly [5]. The S1 subunit is the surface part responsible for binding to the host cell receptor hACE2 (human angiotensin-converting enzyme 2) for viral entry. The S2 subunit is the viral transmembrane part responsible for the fusion of the viral and cellular membranes. The S-proteins form trimeric spikes on the virion surface, which are required for viral entry [6]. On average, there are about 22 N-linked glycans on each S-glycoprotein, which play important roles in protein folding, function, and immune evasion [7,8,9].

Lectins are a large group of natural, bioactive, carbohydrate-binding proteins present in a wide range of organisms, including bacteria, algae, and plants [10,11,12]. In addition to their biological functions, lectins have been found to have potential therapeutic use as anticancer and antimicrobial biomedical agents [13,14,15]. Since lectins are carbohydrate-binding proteins, some of them can recognize carbohydrate structures on the surface of viruses and bind these structures to block viral infections [12,16,17,18]. There are several well-known antiviral lectins, such as cyanovirin-N (CV-N) from cyanobacterium *Nostoc ellipsosporum* [19,20], Griffithsin (GRFT) from the red algae *Griffithsia* [21,22], and Scytovirin (SVN) from cyanobacterium *Scytonema varium* [23,24,25]. They have shown potent activities against a variety of viruses, including HIV, Influenza, and Ebola viruses [26]. Recently, some of these same lectins (CV-N, GRFT), as well as others, have been tested for their antiviral activity against SARS-CoV-2 infection [17,27,28,29,30]. Carbohydrates/glycans, such as mannose (Man) and N-acetyl-d-glucosamine (GlcNAc or NAG), on the surface of virions, are important for the viral structure, immunity, and entry [31,32,33]. If lectins bind to those sugars, the virus can lose its infectivity. Based on this mechanism, lectins should become broad-spectrum inhibitors, which will cover different viruses or different variants of a virus. Moreover, as lectin proteins have immunosuppressive properties, they can be used as microbicides to block infection at the ports of viral entry [34,35]. Therefore, searching for new antiviral lectins is important for antiviral therapeutics.

Extracellular vesicles (EVs) are membrane-enclosed tiny particles containing nucleic acids, proteins, lipids, and metabolites and are generated by all cell types [36,37]. EVs have been found to play important roles in mediating cellular functions and have recently received great attention in biomedical research [36,38,39,40]. EVs are associated with immune response, cancer progression, and viral pathogenicity [41,42,43]. EV-specific surface markers such as CD9, CD63, and CD81 have not been determined for many dietary-derived vesicles, such as those originating from fruits and vegetables, and as such, they are more commonly referred to as vesicle-like nanoparticles (VLNs) [44,45]. In this report, we screened VLNs from a variety of plant and fungal (hereafter broadly referred to as vegetable) sources for antiviral biomolecules such as lectins and miRNA, and we describe the identification of a novel lectin inhibitor from VLNs of shiitake mushroom (*Lentinula edodes*) against SARS-CoV-2, especially the Omicron variant.

## 2. Materials and Methods

### 2.1. Viruses and Cells 

Severe Acute Respiratory Syndrome Coronavirus 2 (SARS-CoV-2, strain USA_WA1/2020, GenBank accession MT020880) was provided by the University of Texas Medical branch. SARS-CoV-2, Omicron variant hCoV-19/USA/GA-EHC-2811C/2021 (Lineage B.1.1.529) was obtained from BEI resources (NR-56481). Vero E6 cells (ATCC^®^ CRL-1586™) were used for neutralization and cytotoxicity assays. HEK-293T cells (ATCC) were used for pseudotyping viruses. Human lung adenocarcinoma epithelial (Calu-3; ATCC HTB-55TM) cells were used for the propagation of infectious SARS-CoV-2 viruses prior to plaque assay neutralization. All cell types were maintained in Dulbecco’s Modified Eagle’s Medium (DMEM) (Gibco™, Grand Island, NY, USA) containing 10% fetal bovine serum (FBS), 8 mM L-glutamine, and 1% Penicillin/Streptomycin (complete DMEM).

### 2.2. Purification of Vegetable VLNs

All vegetable samples were purchased from local grocery stores (Table 1). VLNs were purified from these edible mushrooms and plant species as previously described [44,46,47]. The fresh mushrooms and plants were diced and weighed to a mass of 3 g. The material was resuspended in PBS, minced in a blender, and strained. Next, the samples underwent sequential centrifugation at 500× *g* for 10 min, 2000× *g* for 20 min, and 10,000× *g* for 30 min. The final supernatant was subjected to ultracentrifugation at 100,000× *g* for 2 h. The VLN pellets were washed with PBS and finally resuspended in PBS. The size and concentration of VLNs were determined using a NanoSight NS300 instrument (Malvern, Westborough, MA, USA).

### 2.3. Pseudotyping Viruses

All pseudotyped SARS-CoV-2 viruses were MLV-based and made from pCMV-MLVgagpol and pTG-Luc luciferase reporter plasmids with individual SARS-CoV-2 spike genes, as described by Gary R. Whittaker [48]. The three SARS-CoV-2 variant Spike gene plasmids were purchased from Addgene as follows: pcDNA3.1_spike_del19 (Ancestral, Wuhan Hu-1, MN_908947) [49], pcDNA3.3-SARS2-B.1.617.2 (Delta strain) [50] and pcDNA3.3_SARS2_omicron_BA.1 [51]. Plasmids were transfected into 293 cells seeded in 10 cm^2^ dishes at a ratio of 3:3:4 (MLVgagpol:Spike:Luciferase) using PEI. Forty-eight hours post-transfection, the supernatant was collected, clarified by low-speed centrifugation, and split into single-use aliquots for storage at −80 °C until use.

### 2.4. Pseudovirus Neutralization Assays

Vero E6 cells were seeded in a 96-well plate (8 × 10^4^ cells/well) and incubated in complete DMEM overnight at 37 °C and 5% CO_2_. The following day, serial dilutions of either VLNs or purified recombinant protein were made in complete DMEM at the indicated concentrations and mixed with thawed pseudoviruses. The mixtures were brought to a final volume of 100 µL and kept at room temperature for 1 h. Then, the mixtures were applied to cells in triplicate and returned to the incubator. Six hours later, the virus media was removed, cells were washed with PBS, given fresh, pre-warmed complete DMEM, and returned to the incubator at 37 °C and 5% CO_2_. Two days later, the cells were aspirated, washed once with PBS, and lysed with 1× passive lysis buffer (Promega, Alexandria, Australia). A total of 100 µL of luciferase buffer (Promega) was added to each well, and the relative light units were measured on a luminometer (Veritas, Turner BioSystems, Sunnyvale, CA, USA).

### 2.5. Cytotoxicity Assay of Vegetable VLNs

The MTT-based cytotoxicity assay was conducted as previously described [52] with slight modifications. Vero E6 cells were cultured as described in the neutralization section above. VLNs were diluted to the tested concentrations in complete DMEM and applied to cells in triplicate (100 µL/well). Then, the cells were incubated for two additional days, similar to the neutralization assay. Next, the media was removed, cells were washed with PBS, and 100 µL of a 5 µg/mL MTT solution diluted in optiMEM was added to each well. The cells were returned to the incubator for three hours to allow the purple formalin crystals to form. Afterward, the media was aspirated, and 50 µL of DMSO was added to each well. The plate was subsequently read on a SpectraMax plate reader for absorbance at 570 nm.

### 2.6. Inhibition Analysis of Infectious Viruses by Plaque Assay

Vero E6 cells were seeded in six-well plates at a density of 6 × 10^5^ cells/well and incubated overnight. The Shictin protein was serially diluted in two-fold dilutions and applied to the Vero E6 cells for 4–6 h. After treatment, approximately 100–200 plaque-forming units (PFU) of the virus were added to each well and incubated for 1 h at 37 °C with 5% CO_2_. After viral incubation, the media was replaced, and the cells were covered with an overlay medium containing 1% Sea Plaque Agarose (Lonza). The plates were incubated until plaques were observed on day 3 post-infection, stained with Neutral Red, and counted.

### 2.7. Heat and Sonication of Shiitake Mushroom VLNs

Denaturation of the VLNs was accomplished through either heat treatment or sonication. First, serial dilutions of VLNs were prepared in PBS. Next, one aliquot of the dilutions was transferred to PCR tubes and heated in a SimpliAmp thermocycler for 10 min at 95 °C. A second aliquot was transferred to a microcentrifuge tube and subjected to bath sonication (Branson CPX5800H) at room temperature for 90 min. Afterward, both aliquots were used for neutralization, as described above.

### 2.8. VLNs Evaluation by Scanning Electron Microscopy (SEM)

The untreated, sonicated, and heat-treated VLNs were evaluated by means of SEM, which is an improved and effective method for comparison of size, shapes, and topographic structures of vesicles with and without treatments [53]. Briefly, ~100 µL of VLN suspensions in PBS were collected using a Whatman Nuclepore Hydrophilic Membrane filter (0.05 µm Pore Size), allowing the excess of PBS filtering through the membrane or absorbed by edge-taping with regular filter papers. After 10 min air-dry, the membrane with VLNs was mounted onto the conductive/adhesive tape on SEM stubs. After another short period of air-dry (30–40 min) at room temperature, the samples were sputter coated with a thin layer of chromium (2–4 nm) using the Denton Vacuum Desk V sputtering system. The samples were examined under a Hitachi field emission SEM shortly after coating, and SEM images were collected under comparable conditions.

### 2.9. Proteomics of Shiitake Mushroom VLNs

The proteomics of Shiitake mushroom VLNs were performed as previously described [47]. In brief, the pellets of purified vesicles were lysed and then run on a 12% PAGE for 10 min, followed by in-gel digestion with trypsin. The obtained peptides were subjected to LC-MS/MS analysis. The resulting sequence data were searched against the UniProt database (BL_ProtID_Report_20210117). The identified peptides or proteins were validated using Scaffold v4.8.9 (Proteome Software Inc., Portland, OR, USA).

### 2.10. Molecular Modeling of Shictin

Based on the Proteomic sequence match of A0A1Q3EE58_LENED in UniProt, we searched in GenBank and found it matched the entry of Lectin (*Lentinula edodes*) with the Accession number of GAW05476.1. The full-length structure of Shictin was modeled using the crystal structure of *Streptomyces avermitilis* alpha-l-rhamnosidase (PDB 3W5M) [54] using Modeller [55]. The carbohydrate-binding domain (CBD) structure of Shictin was searched by Phyre2 [56]. The best match is the lectin POL (*Pleurotus ostreatus*) structure (PDB 6T1D) with 44% identity and 100% confidence and other lower homologous protein structures, which are described in the results section.

### 2.11. Recombinant Protein Production

The Shictin gene sequence was codon optimized for *E. coli*, synthesized by GenScript, and cloned into the pET28a vector using BamHI/XhoI restriction enzymes. The plasmid was retransformed into *E. coli* BL-21 DE3(Lys) bacteria and selected using LB media containing 50 µg/mL kanamycin. A total of 10 mM IPTG was added to cultures to induce protein expression overnight at 37 °C. Cell lysis and inclusion body isolation were conducted as previously described [57]. Cells were resuspended in STE buffer containing 100 mM NaCl, 10 mM Tris, 1 mM EDTA, 0.5% NP40, and 100 mg/L lysozyme and disrupted by multiple rounds of sonication. Protein purification and refolding were performed on an AKTApure HPLC machine using a His-trap column. The protein was eluted using 250 mM imidazole and was concentrated and buffer-exchanged using 3 kDa MWCO concentrator spin columns. The final protein was stored in a PBS buffer.

### 2.12. Statistical Analysis

All statistical analyses were conducted using GraphPad Prism (Version 10.0). EC_50_, IC_50_, and CC_50_ values were calculated in comparison with appropriate positive and negative controls using the 4-parameter sigmoidal function in GraphPad Prism. Student’s *t*-test was used to determine significance between treatment groups, with 95% confidence intervals.

## 3. Results

### 3.1. Screening of Vegetable VLNs Inhibiting SARS-CoV-2 Virus Infection

**Figure 1:**
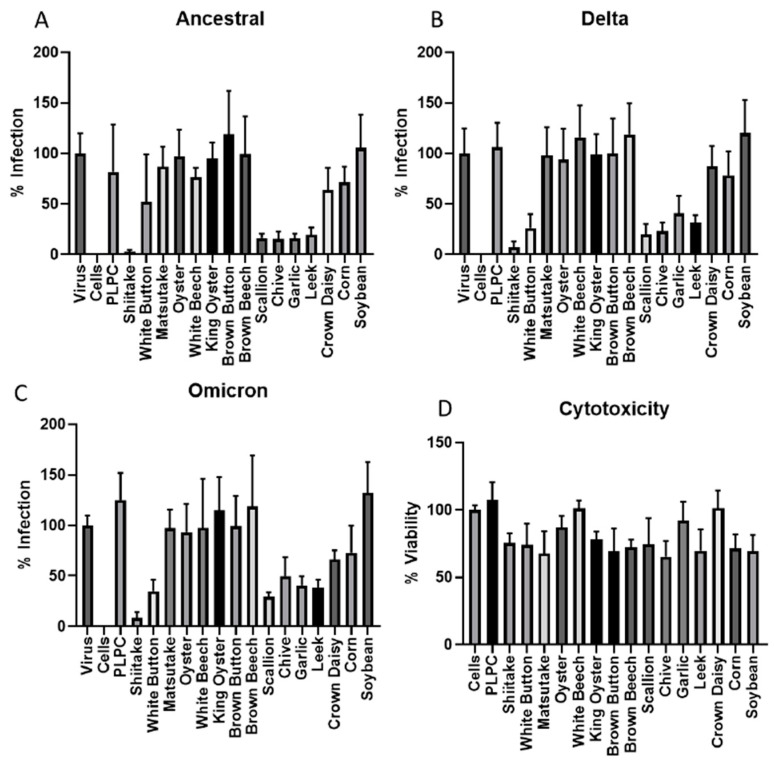
Screening of vegetable VLNs for antiviral activity against SARS-CoV-2 strains. (**A**) Ancestral strain (Wuhan Hu-1). (**B**) Delta variant strain (B.1.617.2). (**C**) Omicron variant strain (BA.1). (**D**) Cytotoxicity assay (MTT) of vegetables VLNs used for antiviral test. PLPC, liposomes used for negative controls.

**Figure 2:**
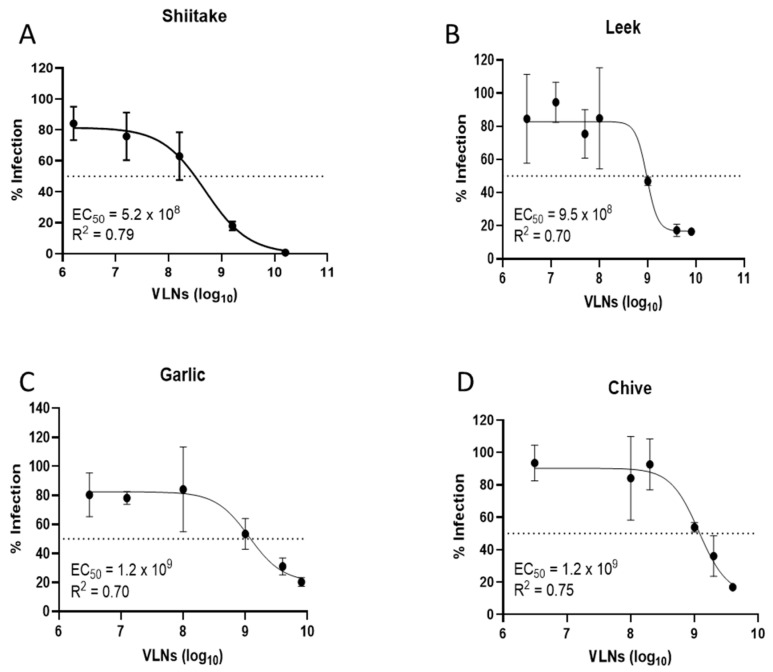
Inhibition assay (EC_50_) of vegetable VLNs against pseudotyped SARS-CoV-2 Ancestral strain (Wuhan Hu-1): (**A**) Shiitake mushroom. (**B**) Leek. (**C**) Garlic. (**D**) Chive.

**Figure 3:**
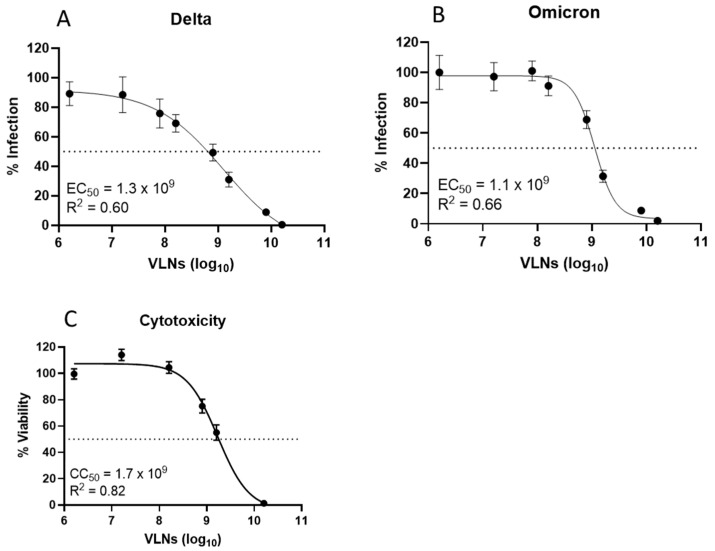
Inhibition assay (EC_50_) of Shiitake VLNs against different pseudotyped SARS-CoV-2 variants: (**A**) Delta variant (B.1.617.2). (**B**) Omicron variant (BA.1). (**C**) Cytotoxicity assay (CC_50_) of Shiitake VLNs.

The VLNs from fifteen local common vegetables were isolated by ultracentrifugation and quantified using a NanoSight N300 instrument (Table 1) [47]. These VLNs were evaluated for their antiviral activities against SARS-CoV-2 infection using an in vitro pseudovirus platform. Three of the major viral variants (Ancestral, Delta, and Omicron) were tested in this screening, and the data are presented in Figure 1A–C. The screening data indicated that shiitake mushroom, white button mushroom, scallion, chive, garlic, and leek-derived VLNs showed better activities against all three SARS-CoV-2 variants. To make sure all these VLNs were not toxic to the cells, we assessed their cytotoxicity using the standard MTT method. The results indicated that all fifteen samples did not show significant cytotoxicity in a concentration of 1 × 10^10^/mL VLNs, with percent viability ranging from 65.3% (chives) to 100% (white beech and crown daisy) (Figure 1D). We further evaluated the antiviral activities of four vegetable VLNs (shiitake, leek, garlic, and chive) in different dosages to determine their EC_50_ values. The results showed that the EC_50_ values were in the range of a few hundred million VLNs, with the shiitake EC_50_ value determined at 5.2 × 10^8^/mL (Figure 2). Since shiitake VLNs exhibited the lowest EC_50_ value of the four tested species, we chose shiitake VLNs for further studies in inhibiting different SARS-CoV-2 variants. The results indicated that it has similar activities against all three variants with EC_50_ values of 5.2 × 10^8^/mL, 1.3 × 10^9^/mL, and 1.1 × 10^9^/mL for Ancestral, Delta, and Omicron variants, respectively (Figure 2A and Figure 3A,B). The cytotoxicity (CC_50_) of shiitake VLNs was also determined, which was 1.7 × 10^9^/mL (Figure 3C). We observed some discrepancies in the toxicity values between the initial screening and the CC_50_ determination, which was likely due to variability between batches of VLNs. However, even the highest calculated EC_50_ value (Delta variant, 1.3 × 10^9^/mL) still had ~60% viability, suggesting that some of the observed neutralization effects can be attributed to biomolecules contained in the VLNs.

### 3.2. Proteins of Shiitake VLNs Contributed to the Antiviral Activities

**Figure 4:**
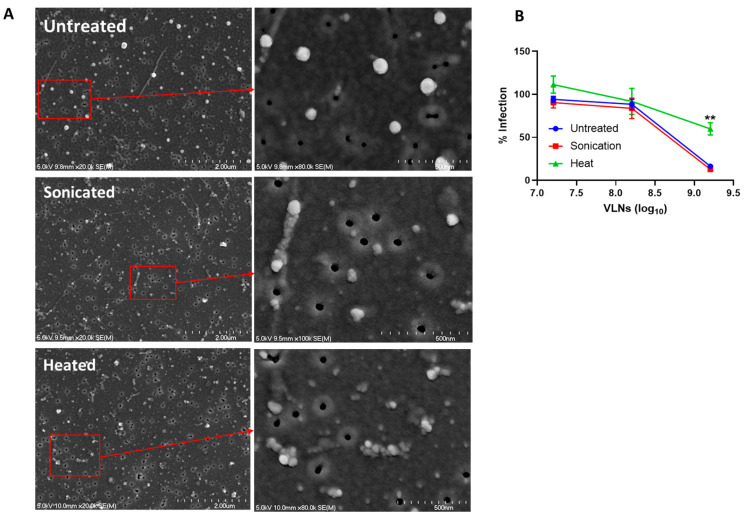
Morphology changes and antiviral function assay of Shiitake VLNs treated by sonication or heat. (**A**) Imaging analysis by Scanning electron microscopy (SEM): Untreated, sonicated, and heated. (**B**) Antiviral activity tests of the treated Shiitake VLNs against SARS-CoV-2 Ancestral strain. The micrographs showing low and high (inserts) magnifications of untreated, sonicated, and heated VLNs. Scale Bars: 2 µm (left panel) and 0.5 µm (inserts). ** *p* < 0.01.

To find out what components in the VLNs may be responsible for the anti-SARS-CoV-2 activity, we conducted biophysical analysis by subjecting shiitake VLNs to sonication and heat treatments. The sonication method works by temporarily creating pores in the VLN membrane, thereby releasing the contents of VLNs, which can then directly interact with the virus for the inhibition assay. The results indicated that sonicated VLNs did show morphological changes, but the inhibition activity remained unchanged (Figure 4A,B). Heat-treated VLNs (95 °C for 10 min) were also morphologically disrupted but showed significantly reduced inhibitory activity (Figure 4A,B). Because heat treatment will denature all proteins, this result suggests that the protein factors in the VLNs of shiitake mushrooms could be associated with the antiviral function.

### 3.3. Lectin (Shictin) of Shiitake VLNs Contributed to the Antiviral Activities

**Figure 5:**
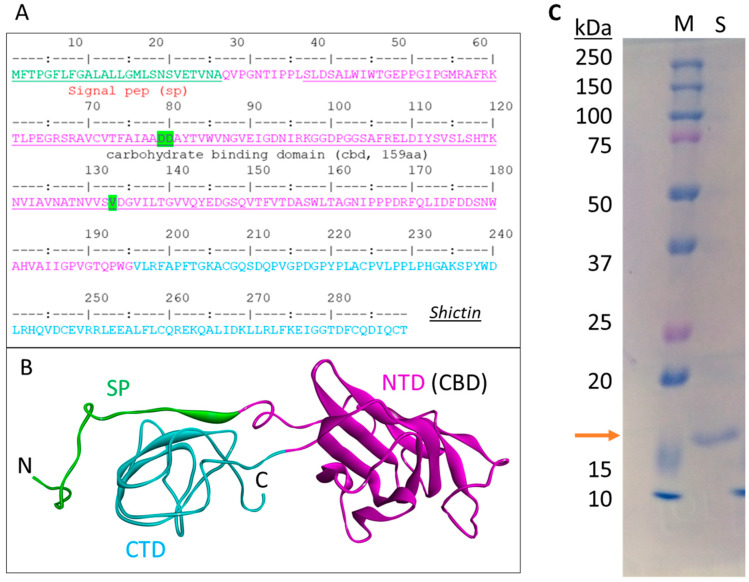
Characterization of Shictin. (**A**) Description of Shictin protein sequence. (**B**) Three-dimensional (3D) structural model of Shictin protein. (**C**) Purified Shictin protein from *E. coli* BL21 cells. SP, Signal peptide (green); NTD, N-terminal domain (magenta); CTD, C-terminal domain (cyan). CBD, carbohydrate-binding domain.

Proteomics was performed to analyze the proteins contained in the shiitake mushroom VLNs. The sequence search against the *Lentinula edodes* transcriptome database found a total of 1655 protein hits, including two lectins-like proteins. Since some lectins have antiviral activities as their carbohydrate-binding properties, we further analyzed these two lectin-like candidates. One hit (A0A1Q3EK95_LENED) is the Clathrin heavy chain, which is the major membrane protein of cytoplasmic vesicles. The other hit (A0A1Q3EE58_LENED) is the lectin protein. Thus, we chose this lectin for further analysis and named it Shictin (Shiitake lectin). This lectin has 289 amino acids consisting of a signal peptide (26aa), a carbohydrate-binding domain (CBD) (159aa) in the N-terminal, which is highly homologous with other known CBDs, a C-terminal domain (94aa), and some short linkers among those segments (Figure 5A,B). From the model of this protein structure based on the carbohydrate-binding module 67 (CBM67) of *Streptomyces avermitilis* alpha-l-rhamnosidase, the CBD is believed to be responsible for the antiviral activity (Figure 5B) [53]. The codon-optimized CBD domain was synthesized and cloned into the pET28a+ protein expression vector. Then, the plasmid of Shictin CBD was transformed into *E. coli* BL21 cells for protein expression and purification. A clear 17 kDa band from purified protein appeared in the 10% PAGE gel, matching the Shictin CBD predicted size (Figure 5C).

**Figure 6:**
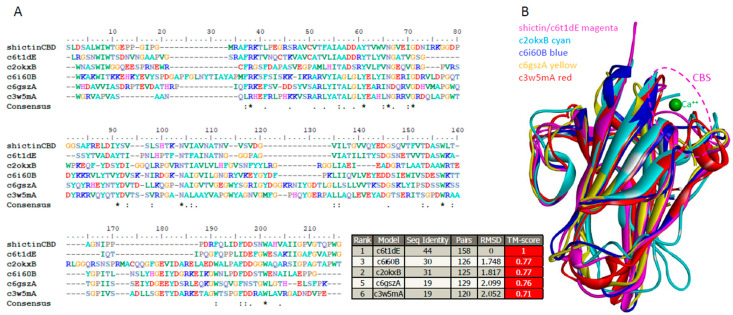
Superposition of five homologous carbohydrate-binding domains (CBDs) with Shictin CBD. (**A**) Protein sequence alignment of homologous CBDs. (**B**) Superimposed homologous CBD structures. The Calcium ion (Ca^2+^) in green is depicted in the carbohydrate-binding site (CBS) [56]. TM-score is a normalized score from 0 to 1, representing overall similarity of the proteins. Scores above 0.5 indicate the same overall fold.

Structural homology analysis found that Shictin belongs to a calcium-dependent sugar-binding family mainly consisting of alpha-l-rhamnosidase and beta-galactosidase. Specifically, Shictin belongs to the non-catalytic domain of the alpha-l-rhamnosidase family. Surprisingly, these lectins have low sequence homology but high structural homology. The five top homologous lectins were compared with their sequences (Figure 6A) and structures (Figure 6B). The sequence identities ranged from 19% to 44%, but their structural identities showed the same folds (a typical β-jellyroll folding) and strong superposition with 100% confidence, RMSD values between 1.748 and 2.099, and TM scores greater than 0.71. This phenomenon demonstrates that the sugar recognition of lectins is not molecule-specific but geometry-specific [58].

**Figure 7:**
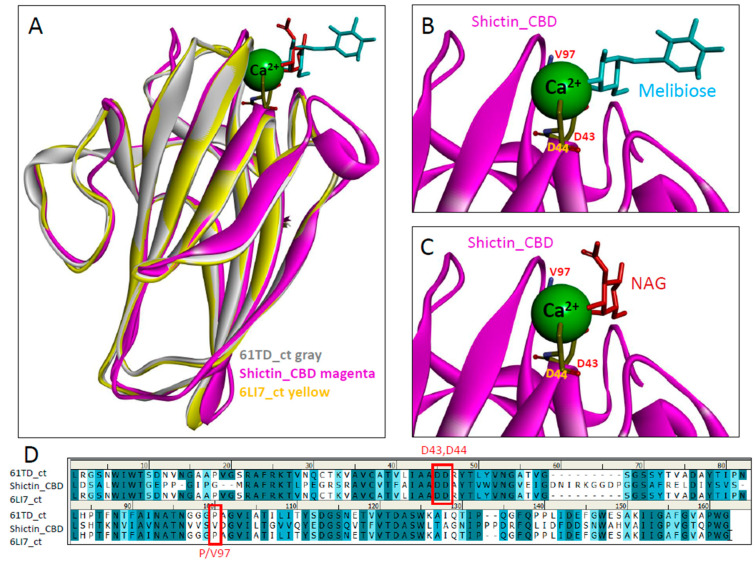
Molecular modeling of carbohydrate-binding domain of Shictin. (**A**) Shictin CBD structural model (magenta) with lectin POL structural models of C-terminal domains (6T1Dct-melibiose, gray-cyan) and (6LI7ct-NAG, yellow-red). (**B**,**C**) Showing the molded carbohydrate-binding of sugar melibiose or N-acetyl-d-glucosamine (NAG) in the carbohydrate-binding site of Shictin CBD. (**D**) Protein sequence comparison of Shictin CBD with lectin POL C-terminal sequences (6T1D_ct) and (6LI7_ct). Three key residues (D43, D44, and P/V97) assumed for sugar-binding are framed in red.

To decipher the sugar-binding of Shictin CBD, we conducted further analysis of the structure and compared it with the highly homologous *Pleurotus ostreatus* Lectin (POL). POL has two carbohydrate-binding domains (N-terminal CBD and C-terminal CBD) containing 42.2% sequence identity, but their structures are very similar, like duplicates [58,59]. In order to compare the 3D structural models, we chose two POL structures, which are solved with different sugars, melibiose (PDB 6T1D) [59] and N-acetyl-d-glucosamine (PDB 6LI7) [58]. Shictin CBD was superimposed with these two CBD structures (Figure 7A), and the calcium-dependent sugar-binding was modeled in the carbohydrate-binding site (Figure 7A–C). Two Aspartic acid residues (D43 and D44) are conserved for calcium-based sugar (melibiose or NAG)-binding in the carbohydrate-binding sites (Figure 7B or Figure 7C). Another residue, Valine (V)97 in Shictin but Proline (P) in lectin POL, also contributes to carbohydrate-binding in this binding site, as determined from the sequence comparisons (Figure 7D). The sequence differences in this position of P/V97 may be associated with their sugar-binding and functional differences [60].

### 3.4. Shictin Inhibits SARS-CoV-2 Infection

**Figure 8:**
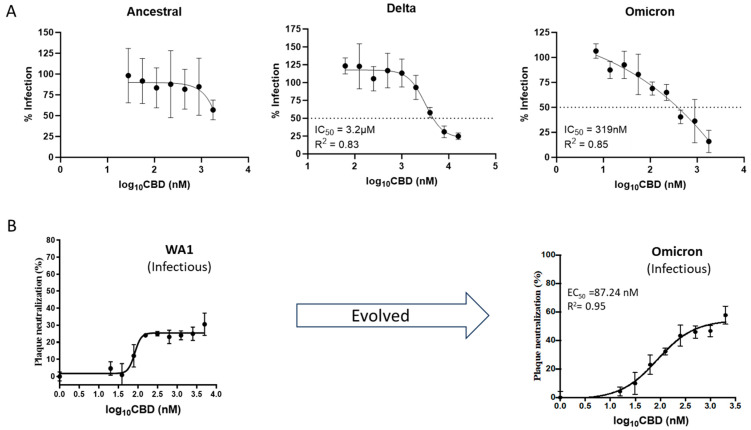
Shictin (CBD) inhibition assays (IC_50_) against SARS-CoV-2 variants. (**A**) Inhibitions against pseudotyped viruses [Ancestral (Wuhan Hu-1), Delta (B.1.617.2), and Omicron (BA.1)]. (**B**) Inhibitions against infectious viruses [Ancestral (WA1) and Omicron (B.1.1.529)].

Purified Shictin-CBD protein was evaluated for its antiviral activity against three pseudotyped SARS-CoV-2 variants in vitro. The data show that Shictin had potent activity against the Omicron variant, with an IC_50_ value of 319 nM, but limited-to-moderate activity against the Ancestral and Delta variants (Figure 8A). To confirm the pseudovirus neutralization data, we conducted plaque reduction neutralization assays using the Shictin-CBD protein against two different strains of infectious SARS-CoV-2 virus: the US Washington strain (WA1) and the Omicron variant (B.1.1.529). The results indicate that the pseudovirus data are analogous to the infectious virus data. The WA1 strain, which is closely related to the Wuhan ancestral strain, showed limited activity. However, for the Omicron strain, the IC_50_ value was 87.24 nM, which is comparable to the corresponding pseudovirus result (Figure 8B). Taken together, the inhibition data suggest that Shictin is especially potent against the SARS-CoV-2 Omicron variant.

## 4. Discussion

Exosomes or EVs play an essential role in cell functions and are also important for therapeutics. Therefore, the composition of exosomes has been extensively investigated. In this research, we found that lectin proteins can be incorporated into vegetable vesicle-like nanoparticles, which is informative for finding lectins. In our screening assay, we observed some fungal and plant VLNs that exhibited antiviral activity. From VLNs of Shiitake mushrooms, we identified an antiviral lectin (Shictin). Our data suggest that Shictin contributes to the antiviral activity of Shiitake mushroom VLNs, but there may be other components that also play a role in this activity. Thus, it is possible to develop VLN-based antiviral therapeutics.

Interestingly, Shictin showed a striking difference in neutralizing the three tested variants of SARS-CoV-2. Based on the number of predicted N-glycosylation sites on the spike protein, total glycan numbers should not account for the neutralization differences, as the Ancestral and Omicron strains have 22, while Delta has 21. Thus, the difference in Shictin inhibition might instead be due to differences in carbohydrate composition, site occupancy, and glycosylation patterns among variants. In addition, local amino acid sequence variations around N-glycosylation sites also influence the glycosylation type, length, and efficiency, potentially affecting the Shictin-binding affinity and neutralization activity. More interestingly, we noticed that the inhibition potency increased during the evolution of the virus, from Ancestral (weak), to Delta (moderate, IC_50_ = 3.2 µM), to Omicron (strong, IC_50_ = 87 nM). This may be associated with the glycosylation changes on the virion surface. A recent report on analysis of S-glycoprotein glycosylation of SARS-CoV-2 variants indicated that from Alpha to Delta, there was a decrease in the overall abundance of complex-type glycans coupled with an increase in the oligomannose-type glycans. However, Omicron contains fewer oligomannose-type glycans compared to Delta but does contain more compared to the wild-type [61,62]. That may explain Shictin neutralization data, as these different patterns of glycosylation affect the binding affinity. Glycosylation changes play an important role in viral variation and evolution and, consequently, become the variants of concern (VOCs) of SARS-CoV-2 [62,63]. Furthermore, viral entry pathways may also affect Shictin neutralization differences between strains. There are two distinct pathways for SARS-CoV-2 entry into cells: endosomal entry utilizing ACE2/cathepsin-B/L and TMPRSS2-dependent cell surface entry [64,65]. SARS-CoV-2 Omicron variants have evolved to have reduced TMPRSS2 usage and increased hACE2 fusogenicity, leading to endosomal entry [66,67]. The Vero-E6 cells used in this study notably lacked TMPRSS2, so the SARS-CoV-2 virus could not use the cell membrane fusion pathway [68], which may explain Shictin’s stronger potency against Omicron. Because Shictin likely binds mannose residues on the viral spike protein and, therefore, interferes with ACE2-binding, it is likely more sensitive against Omicron in this cell type since Omicron cannot enter cells through the endosomal pathway.

A structural search of Shictin found the top hits are alpha-l-rhamnosidases, which belong to the CBM67 family [54]. They are the non-catalytic domain of alpha-l-rhamnosidases. This carbohydrate-binding module (CBM) is calcium-dependent, and sugar-binding; the two conserved aspartic acids (D43 and D44) are important for interacting with the Ca^2+^ ion for sugar-binding.

Lectin proteins can be isolated from fruiting bodies of mushrooms, such as lectin LEL from shiitake mushroom (*Lentinula edodes*) [69], lectin POL from oyster mushroom (*Pleurotus ostreatus*) [59], and lectin HSL from Agaricomycetes [70]. Our study demonstrates that lectin proteins exist in vesicle-like nanoparticles, which provides a valuable source for identifying novel lectins in biomedical research. Certainly, the exosomes (VLNs) from fungi or plants will be helpful for finding antiviral lectins and especially for the development of broad-spectrum antiviral agents.

## Figures and Tables

**Table 1:** List of vegetable VLNs evaluated.

No	Name	Concentration (/mL)
1	Shiitake mushroom (*Lentinula edodes*)	1.05 × 10^13^
2	White button mushroom (*Agaricus bisporus*)	3.15 × 10^12^
3	Matsutake mushroom (*Tricholoma matsutake*)	2.3 × 10^12^
4	Oyster mushroom (*Pleurotus ostreatus*)	5.0 × 10^12^
5	White beech mushroom (*Hypsizygus tessellatus*)	7.0 × 10^12^
6	King Oyster mushroom (*Pleurotus eryngii*)	1.3 × 10^13^
7	Brown button mushroom (*Agaricus bisporus*)	2.65 × 10^12^
8	Brown beech mushroom (*Hypsizygus tessellatus*)	6.5 × 10^12^
9	Scallion (*Allium fistulosum*)	2.2 × 10^12^
10	Chive (*Allium tuberosum*)	8.0 × 10^12^
11	Garlic (*Allium sativum*)	2.6 × 10^12^
12	Leek (*Allium ampeloprasum*)	2.6 × 10^12^
13	Crown daisy (*Glebionis coronaria*)	3.6 × 10^12^
14	Sweet corn (*Zea mays*)	1.45 × 10^12^
15	Soybean (*Glycine max*)	6.0 × 10^12^

## Data Availability

Data will be made available on request.
